# Increased Liveliness of Trunk Muscle Responses in Elite Kayakers and Canoeists

**DOI:** 10.3390/sports8060078

**Published:** 2020-05-29

**Authors:** Andrej Kocjan, Nejc Šarabon

**Affiliations:** 1 Faculty of Education, University of Primorska, SI-6000 Koper, Slovenia; andrej.kocjan@pef.upr.si; 2 Faculty of Health Sciences, University of Primorska, SI-6000 Koper, Slovenia; 3 Department of Health Study, University of Primorska, Andrej Marusic Institute, SI-6000 Koper, Slovenia; 4 Laboratory for Motor Control and Motor Learning, S2P Ltd., SI-1000 Ljubljana, Slovenia

**Keywords:** exercise, strength, stability, low back pain

## Abstract

Trunk stability functions play an important role in sport and everyday movements. The aim of this study was to analyze trunk strength, trunk muscles onset of activity, and rate of electromyographic rise (RER) in the case of self-inflicted and unexpected trunk loading. Thirty-two healthy young adults (16 elite kayakers/canoeists and 16 non-athletes) were measured with a multi-purpose diagnostic machine. Trunk strength was assessed in standing position. Trunk muscles onset of activity and RER were assessed through unexpected loading over the hands and rapid shoulder flexion, respectively. In comparison with non-athletes, kayakers/canoeists did not significantly differ in trunk strength and showed lower trunk extension/flexion strength ratio (*p* = 0.008). In general, trunk muscles onset of activity did not significantly differ between the groups. On the contrary, kayakers/canoeists showed higher RER mean values in all the observed muscles (*p* < 0.041), except in multifidus muscle during self-inflicted movements. Similarly, higher RER variability was observed in the majority of the observed muscles among kayakers/canoeists. Higher RER among kayakers/canoeists could represent a protective mechanism that ensures spine stability and prevents low back pain.

## 1. Introduction

Elite athletes who compete in agility-based sports demonstrate greater strength and power outputs than endurance trained athletes or non-athletes [1]. Kayaking/canoeing is an upper body sport where the result is largely affected by paddlers’ ability to control four boat movements (center bouncing, end bouncing, snaking, and rocking) [2]. The efficiency of paddling and balancing during unstable sitting/kneeling largely depend from trunk strength and stability. Both mentioned abilities are functionally important because of the energy transfer from the trunk to lower and upper extremities. Inappropriate inter-muscular coordination (in temporal, intensity, or special domain of muscle activation), due to impaired trunk muscles onset of activity or rate of torque development, can result in poor energy transfer between the lower and upper extremities or spinal injury [3]. Furthermore, an adequate strength ratio between trunk extensors and flexors prevents future low back pain (LPB) [3], which is increasing among young athletes and adolescents [4].

The level of spinal stability depends on several factors. One of those is neuro-muscular control [5]. Anticipatory postural adjustments (APAs) and proper postural reflex responses (PRRs) of trunk muscles are important to control the position of facet joints [6,7]. Trunk muscles activity, which affects proper vertebral alignment during APAs, is pre-programmed by the central nervous system and occurs prior to self-induced perturbations [8]. Hodges and Richardson [9] demonstrated that the onset of electromyographic (EMG) activity of several superficial trunk muscles during self-induced perturbations varied according to the movement direction and its velocity. Furthermore, very stable [7] and very unstable [10] surfaces result in decreased EMG amplitudes during self-induced rapid movements. Several studies showed that APAs improved after exercise. A single session of medicine ball throws resulted in improved onset of activity of trunk muscles during rapid shoulder flexion maneuver [11]. Tsao and Hodges [12] demonstrated improved trunk muscles onset of activity after a single session of deep abdominal muscle training. In contrast with a stable environment, exercises performed on unstable surfaces showed greater improvement of muscles onset of activity during APA experiments [13,14].

When a sudden perturbation occurs to the spine, PRRs provide the first line of defense to ensure trunk stability [15]. Altered trunk muscles’ shut-off and/or switch-on latency during unexpected perturbations were shown in LBP patients [16,17] and athletes with a history of LBP [18] and were proposed as a risk factor in future low back injury in athletes [5]. Moreover, in comparison with healthy subjects, LBP patients showed increased variability of muscles onset of activity during unexpected trunk loading [19]. The efficiency of PRRs largely depends on stretch reflex, which can be modified with training [20]. Balance training proved to be an effective method to improve trunk muscles EMG reaction time in healthy subjects [21] and LBP patients [14,22].

In addition to quick onset of muscles activity, the rate of force development provides an important basis for spinal stability [23]. The rate of EMG rise (RER) is one of the key determinants of the early phase (25–75 ms after the onset of muscular contraction) of the rate of force development [24] and is strongly influenced by exercise. Vila-Cha et al. [25] showed that RER improved by 80% after 6 weeks of high intensity strength training, while it did not change after moderate intensity cycling.

The knowledge about the effectiveness of long-term training on trunk muscles onset of activity and RER the in case of unexpected and expected trunk loading in elite athletes is very limited. To the authors knowledge, there is no study that has investigated the effect of combined trunk sensory-motor training and upper body resistance exercise, such as in the case of kayaking and canoeing, on trunk muscles RER in elite athletes. In the present study, we tested the hypothesis that long-term wild water kayak/canoe practice and associated strength and conditioning training increases trunk strength and trunk muscles onset of activity and RER in the case of self-inflicted and unexpected trunk loading.

## 2. Materials and Methods

### 2.1. Experimental Approach to the Problem and Study Design

The study was cross sectional, where we investigated trunk stability of elite kayakers/canoeists and physically inactive subjects. We hypothesized that long-term wild water kayak/canoe practice increases strength of trunk muscle groups as measured per trunk maximal isometric voluntary contractions (MIVC) in frontal (lateral bending) and sagittal planes (flexion and extension) and improves trunk stability responses. Trunk stability was assessed via muscles onset of activity and RER of several trunk muscles, triggered by self-inflicted and unexpected external loading.

### 2.2. Subjects

Thirty-two healthy young adults participated in the study. Sixteen subjects, members of the Slovenian national wild water kayak and canoe team (kayakers/canoeists), with a minimum of 4 years of regular training (average 3 times per week) and sixteen subjects without regular physical activity (non-athletes) took part. Inclusion criteria were controlled via self-reported questionnaire and were confirmed by athletes’ coaches. Eleven kayakers/canoeists had history of LBP. The structure of the participants, including age, gender, body mass, and body height is presented in Table 1. Subjects were informed about the study protocol before the beginning of the experiment and confirmed voluntary participation by signing the informed consent. The study was approved by the National Medical Ethics Committee (107/011-12).

### 2.3. Procedures

#### 2.3.1. Measurement Techniques

**Figure 1:**
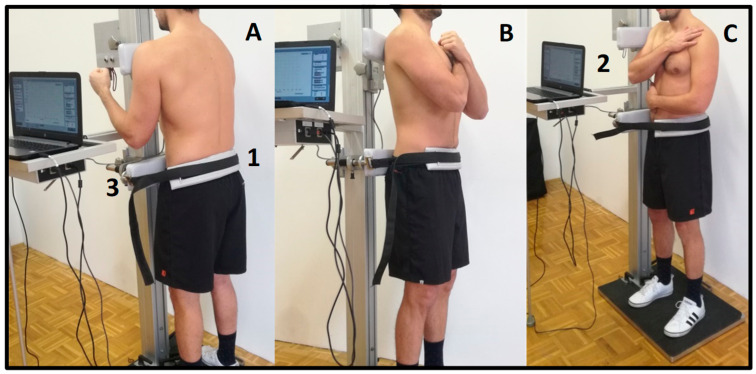
Measurement setup for trunk strength assessment. Measurement setup for trunk flexion (**A**), extension (**B**), and trunk lateral flexion (**C**) isometric strength testing. The strap across the pelvis (1) provides good body fixation. The height of the upper bar, which incorporates the force sensor (2) and the lower bar (3), can be regulated manually according to subjects’ morphological characteristics. The vertical center-to-center distance between the two bars represented the lever arm, used for torque calculation in the maximal isometric voluntary contractions trials.

A commercial multi-purpose trunk diagnostic machine (TNC system, S2P Ltd., Ljubljana, Slovenia) was used to measure trunk isometric strength (Figure 1) and trunk stability through APA and PRR. The machine incorporates the force sensor (Z6FC3—200 kg, HBM, Darmstadt, Germany) as a part of the isometric dynamometry module, an EMG module, and an electro-magnetic quick release module. Participants performed a warm-up (20 times alternating high knee lifts, 10 push-ups, 10 overhead squats, 20 alternating single leg dead lifts). Both support bars (height = 10 cm, width = 45 cm) were rigid padded elements and were adjusted manually in order to meet individual anthropometric characteristics. The upper edge of the machine’s lower support bar was positioned at the level of anterior superior iliac spine and was horizontally adjusted to assure the neutral spine position during the MIVC task. The pelvis was firmly tightened to the lower support bar using a rigid padded strap (width = 5 cm). The upper support bar that was in contact with the subject’s body at the shoulder’s height, was equipped with a compression load cell (Z6FC3—200 kg, HBM, Darmstadt, Germany). The subjects were instructed to perform three MIVC in each direction (trunk extension, flexion, and lateral flexion to the right) in standing position with arms crossed on the chest. All subjects were instructed to maximally press with the upper body against the sensor-equipped bar while keeping the knees extended and ankles underneath the hips to prevent from pushing with the legs. Thus, the subject’s position relative to the dynamometer was: (i) back contact for trunk extension MIVC, (ii) front contact for flexion MIVC, and (iii) side contact for lateral flexion MIVC. A single muscle action gradually increased over ~2 s, followed by ~3 s of MIVC. Rest periods between individual muscle actions were ~20 s long, while rest periods between strength tests of different muscle groups were ~1 min. All subjects were verbally encouraged to exert their maximal effort. The signal was 400× amplified, analog-to-digital converted, and acquired at a sampling rate of 1000 Hz (NI-USB-6009, NI, Austin, TX, USA). The signals were stored on a personal computer for later analysis.

**Figure 2:**
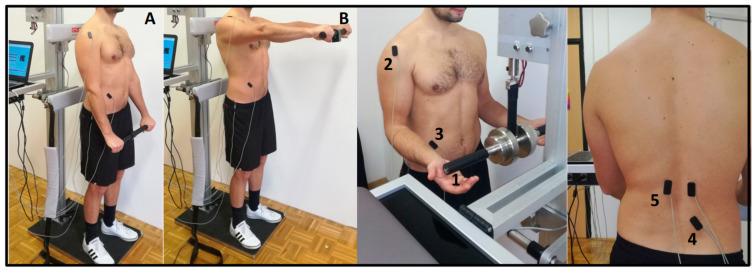
Measurement setup for testing trunk stability functions. Anticipatory postural adaptations were assessed through rapid shoulder flexion maneuver. The subjects’ task was to stand relaxed (**A**) and flex the shoulders to 90° (**B**) as fast as possible—self initiated in 2-s time intervals after the acoustic signal. Postural reflex responses were assessed through unexpected external trunk loading over the hands. Subjects gently touched the handle (1) of the loading system and waited for a sudden release of 8% body mass load. The subjects’ task was to stop the load as soon as possible after its automatic release. During both stability tasks, electrical activity of anterior deltoid (2), obliquus externus (3), and multifidus (4) muscle on the right and erector spine (5) muscle bilaterally were recorded.

The data on trunk muscles PRRs were provided by the system which enables unexpected external loading over the hands [26] (Figure 2). Subjects gently grabbed the handle of the loading system and waited for a sudden load (8% of body mass) release, which randomly occurred every 6 to 12 s. The position of the loading system was adjusted in vertical direction to provide 90° of elbow flexion. All subjects were instructed to sustain their normal body posture (feet parallel at hips width) and to stay relaxed. The subjects’ task was to stop, i.e., stabilize the load as soon as possible after its automatic release, hold it for ~1 s, and then reattach it into the initial position.

To obtain the data from trunk muscles APAs, rapid shoulder flexion maneuver was used. All subjects handled a stick (300 g) which incorporated an accelerometer and were instructed to stand relaxed with feet parallel at hip width and arms extended in front of the hips. The subjects’ task was to flex the shoulders to 90° as fast as possible after ~2 s, following the acoustic signal, which randomly occurred between every 6 to 12 s. After rapid shoulder flexion and ~1 s of holding the accelerometer at eye level, the subjects slowly returned it into the initial position and waited for the next acoustic signal. In both PRR and APA, the subjects performed 2 sets of 10 repetitions (~1 min rest between sets).

Electrical activity of trunk muscles was measured with surface EMG. EMG signals were 1500-times amplified (INA121UA, Texas Instruments, Dallas, TX, USA; input resistance 1 TΩ) and captured via an analog-digital card (NI USB-6343, National Instruments Inc., Austin, TX, USA) with a frequency of 4000 Hz. An anti-aliasing analog filter was implemented using a low-pass filter with a bandwidth of 1 kHz. Signal acquisition and processing was done by custom software (Labview, National Instruments Inc., Austin, TX, USA). All signals were displayed in real time and stored in a personal computer for further analysis. In order to measure muscle activation during both stability tasks, we used the bipolar setup of surface EMG pairs of electrodes (diameter = 10 mm, center-to-center distance = 20 mm, zinc cup electrodes filled with Ten20 conductive paste (Weaver and Company, Denver, CO, USA). A pair of electrodes were embedded in a thin plastic plate, enabling good fixation to the body using two-sided adhesive tape and placed in accordance with previous studies [27] on obliquus externus (OE) (positioned at 45° above anterior superior iliac spine in line with the umbilicus), multifidus (MF) (positioned lateral to spinous process of L5, on the line which connects posterior superior iliac spine and spinous process of Th12), anterior deltoid muscle on the right side (positioned 2 cm distal and anterior to the acromion, on the line which connects the acromion and the thumb) on left erector spinae (ESL) and on right erector spinae (ESR) (positioned vertically 2 cm lateral to spinous process of L1). Electrode position of MF could be questionable because of potential crosstalk with lumbar longissimus [28]. A 5 × 5 cm square self-adhesive electrode (STIMEX, Pierenkemper GmbH, Wetzlar, Germany) was placed over the great femur trochanter as the ground electrode. To prevent mechanical artefacts in the EMG signals, we added an additional strap of surface tape over the wire and the electrode. At the locations of the electrodes, the skin was previously abraded with Nuprep gel (DO Weaver & Company, Aurora, CO, USA) to reach skin resistance <5 kΩ, cleaned with medical alcohol and dried in order to assure good contact and fixation.

#### 2.3.2. Signal Processing and Statistical Analysis

Custom developed software (ARS-Trunk, S2P Ltd., Ljubljana, Slovenia) was used for signal post-processing. For MIVC assessment, only the repetition with the highest mean force output within 1 s time interval was included into the further analysis. The vertical center-to-center distance between the two bars represented the lever arm, used for torque calculation in the MIVC trials (Torque (Nm) = Force (N) × Lever arm (m)). The onset of trunk muscles activity during PRR and APA analyses were defined by a computer protocol and represented the point when the target muscle EMG amplitude increased by two standard deviations above the baseline value [29]. The latter was defined as a 50 ms window before the onset of deltoid muscle activity for APA and the 50 ms window before the release of the loading system for PRR analysis. RER was defined as a percentage of peak amplitude (%/s) of a single task at 50 ms after trunk muscles onset of activity. In the case of APA and PRR analysis, the EMG signals were filtered (Butterworth band-pass, 3 to 500 Hz, level 2, zero phase shift) and smoothed (moving window root-mean-square, 20-ms window), followed by a linear envelope calculation (Butterworth 10 Hz low-pass filter, level 2, zero phase shift) (for details see [26]).

The normality of distribution and homogeneity of variances of all outcome variables were verified by Shapiro–Wilk and Levene’s test, respectively. In the case of normal distribution, the T-test for independent samples was used to test the differences between the two groups of subjects. When variables were not normally distributed, we used a nonparametric, Mann–Whitney test. To avoid type I error, we corrected p-values for multiple pairwise comparisons using the Benjamini–Hochberg correction method. Results (mean and variability) are reported as mean ± standard deviation. The level of statistical significance was set to *p* < 0.05 and effect size (ES) estimation (r^2^, η^2^) is presented. Statistical analyses were done in SPSS (SPSS statistics 19, IBM, New York, NY, USA).

## 3. Results

All results are presented in Table 2. In comparison with non-athletes, kayakers/canoeists showed tendency of higher torque values in direction of trunk flexion and lateral flexion. In direction of trunk extension, torque values were not significantly different between the two groups. Consequently, the strength ratio between trunk extensors and flexors was higher among non-athletes.

In the case of PRR, mean and variability values of trunk muscles onset of activity did not significantly differ between kayakers/canoeists and non-athletes in any of the observed muscles. In contrast with muscles onset of activity, mean values of RER and variability of RER differ significantly between the groups among all muscles, except in variability of MF.

In the case of APA, mean values of trunk muscles onset of activity did not significantly differ between kayakers/canoeists and non-athletes in any of the observed muscles except ESR. Variability of trunk muscles onset of activity was significantly higher among non-athletes in ESR while MF, ESL, and OE did not significantly differ between the groups.

RER mean values were significantly higher among kayakers/canoeists in all observed muscles except in MF. Similarly, kayakers/canoeists showed higher RERvariability in all observed muscles except in ESR and MF.

## 4. Discussion

The purpose of our study was to investigate trunk strength and trunk stability functions between elite kayakers/canoeists and physically inactive subjects. We hypothesized that in comparison with physically inactive subjects, kayakers/canoeists will express greater trunk strength output, earlier muscles onset of activity, and higher RER during APA and PRR tests. The results only partly support our hypothesis. Elite kayakers/canoeists showed significantly higher RER mean values (except MF during APA analysis). Trunk strength and trunk muscles onset of activity did not significantly differ between the groups.

Kayakers/canoeists who participated in our study performed on average 11 training sessions per week and did not significantly differ in maximal torque values from non-athletes. These surprising results could be explained in several ways. The time that paddlers need to complete their racing course indicates that wild water kayaking/canoeing has an important aerobic component. Training that produces muscle fatigue favors slow muscle fibers, which could affect maximal torque production. A history of LBP, which was present in the majority of our kayakers/canoeists, could represent another trigger for a decreased peak torque outcome. Hodges and Richardson [30] demonstrated that healthy subjects with LBP history had impaired neuro-muscular function even in the absence of pain. Moreover, a fear of pain, which typically occurs in the case of spinal loading during strength testing, could be another factor for insignificant differences in maximal strength. Nevertheless, kayakers/canoeists’ extension/flexion strength ratio of 1.2 still represents a “safe zone”, which is typical for healthy subjects [3,31].

Trunk sudden loading showed that kayakers/canoeists had 13.3%/s to 9.3%/s higher RER of back muscles than non-athletes. The latter finding is in accordance with the literature, where investigators demonstrated greater neural excitability during the early phase of rate of force development after high intensity strength training [32]. Besides MF that was extensively studied in the context of spinal stability [17,33], also ESL and ESR have an important function in providing spine stability. Especially spinalis and longissimus muscles with their direct vertebral attachments provide an important vertebral fixation during unexpected trunk loading. Furthermore, the interpretation of the results regarding MF is difficult because the potential cross-talk of lumbar longissimus.

To the authors knowledge, there has been no study that demonstrated improved muscles onset of activity after exercise in healthy adults [34]. Similar results were found in our study, where muscles onset of activity did not differ between the two groups. Trunk position during rowing and kayaking increased posterior pelvic tilt, which tensely loaded active and passive tissues on the posterior side of the lumbar spine. Decreased sensitivity of muscle spindles could result in decreased muscles onset of activity [35] and decreased amplitudes [36] of back muscles PRR. Furthermore, Hendershot et al. [37] found that prolonged exposure to trunk flexion resulted in lower stiffness of passive vertebral structures during trunk sudden loading applied in standing position (neutral spine alignment). Consequently, the reflexive onset of activity of back muscles could be delayed because of an excessive ligamental laxity [38,39]. In the latter case, high RER among kayakers/canoeists could represent a compensatory mechanism of decreased stiffness of passive tissues. Therefore, high RER could be the consequence of several factors. Miller et al. [40] found that LBP patients had delayed muscles onset of activity after sudden trunk loading as a result of endurance training, which could represent another trigger for higher RER (compensatory mechanism) among kayakers/canoeists. The inability to provide muscle activation early enough could be compensated with greater force gain. Higher RER, which is the most dominant factor of rate of force development during first 100 ms after the onset of muscle contraction, could be a possible mechanism to provide a vertebral fixation on time. It seems that an adequate level of spinal stability could be obtained also with a powerful activation, rather than a faster onset of muscular activity. As expected, the smallest difference in PRR RER between the groups was observed in OE, where kayakers/canoeists showed a slightly higher value (5.8%/s). The latter could be the consequence of our trunk loading system that produces trunk perturbation in direction of trunk flexion, which does not stimulate stretch reflex of trunk flexors. Moreover, OE did not have direct vertebral attachments and is not the prime muscle to ensure the rise of intra-abdominal pressure. Both factors suggest that OE has a minor role in providing spinal stability.

In comparison with mean values, a variability of RER and muscles onset of activity is not well understood. Our results showed that kayakers/canoeists had significantly higher RER variability in the majority of analyzed muscles. Pain [41], nerves damage [38], and the loss of type II fibers [42] among LBP subjects, could be possible causes that contribute to greater variability. Furthermore, patients with LBP expressed greater co-contraction [43] of some trunk muscles during sudden trunk unloading, which indicates impaired intra-muscular coordination—another potential factor for a higher RER variability among kayakers/canoeists.

Our study has several limitations which relate to group homogeneity and familiarization with the testing protocol. The majority of kayakers/canoeists tested in our study had a history of LBP, which could result in decreased torque output and delayed reflex response. The subjects did not undergo a separate preliminary learning visit (familiarization with the equipment and testing protocol), which could influence lower reliability of outcome force values.

Higher RER among kayakers/canoeists could represent a protective mechanism that ensures spine stability and prevents LBP. Unstable sitting with additional pulling exercises should be a subject for future research due to its potential to develop proximal stability in young adults. Despite the onset of muscles activity, future research should investigate how fast muscles develop strength in conditions of unexpected trunk loading.

## Figures and Tables

**Table 1:** Basic parameters (social and anthropometry) of the subjects.

	All	Kayakers/Canoeists	Non-Athletes	T-Test (p)
N All (male/female)	32 (24/8)	16 (12/4)	16 (12/4)	
Age (years)	22.2 ± 4.6	21.0 ± 4.0	23.3 ± 5.3	0.176
Body height (cm)	177.0 ± 9.2	174.7 ± 8.9	179.3 ± 9.5	0.121
Body mass (kg)	73.0 ± 12.1	70.6 ± 10.6	75.5 ± 13.5	0.262

**Table 2:** Comparison of strength and stability functions between kayakers/canoeists and non-athletes.

		Kayakers/Canoeists (M ± SD)	Non-Athletes (M ± SD.)	*p*-Value (ES)
**STRENGTH** **(Nm)**	FLEX	670.1 ± 204.4	512.5 ± 197.8	0.077 (0.14)
	L_FLEX	661.0 ± 144.0	518.5 ± 197.1	0.076 (0.16)
	EXT	794.3 ± 158.8	688.0 ± 212.4	0.416 (0.03)
	E/F RAT	1.2 ± 0.2	1.3 ± 0.3	0.008 * (0.25)
**PRR_LAT_M** **(ms)**	ESL	115.4 ± 10.1	118.7 ± 9.9	0.450 (0.03)
	ESR	113.8 ± 9.2	118.7 ± 9.0	0.215 (0.07)
	MF	113.9 ± 9.5	119.7 ± 9.7	0.169 (0.09)
	OE	130.9 ± 13.3	124.9 ± 14.3	0.979 (0.001)
**PRR_LAT_SD** **(ms)**	ESL	10.7 ± 2.8	14.9 ± 13.6	0.963 (0.00)
	ESR	9.9 ± 3.2	13.8 ± 10.4	0.172 (0.00)
	MF	11.1 ± 3.1	15.5 ± 10.1	0.167 (0.00)
	OE	13.3 ± 7.3	19.4 ± 14.3	0.560 (0.01)
**PRR_RER_M** **(%/s)**	ESL	19.0 ± 6.6	5.6 ± 2.1	<0.001 * (0.72)
	ESR	15.3 ± 5.9	6.0 ± 1.4	<0.001 * (0.60)
	MF	20.9 ± 9.3	10.3 ± 4.5	0.003 * (0.33)
	OE	9.6 ± 6.1	3.8 ± 2.6	<0.001 * (0.59)
**PRR_RER_SD** **(%/s)**	ESL	7.3 ± 3.7	2.2 ± 1.3	<0.001 * (0.57)
	ESR	5.2 ± 2.0	2.4 ± 1.4	<0.001 * (0.41)
	MF	5.4 ± 2.7	4.2 ± 3.7	0.185 (0.08)
	OE	3.8 ± 3.1	0.8 ± 0.6	<0.001 * (0.60)
**APA_LAT_M** **(ms)**	ESL	3.8 ± 10.0	−5.5 ± 14.6	0.084 (0.13)
	ESR	−0.5 ± 9.5	−0.3 ± 12.1	0.971 (0.00)
	MF	−2.3 ± 11.7	0.0 ± 13.0	0.712 (0.01)
	OE	35.0 ± 27.3	37.5 ± 20.4	0.843 (0.00)
**APA_LAT_SD** **(ms)**	ESL	16.2 ± 7.2	21.4 ± 10.5	0.191 (0.08)
	ESR	13.3 ± 4.1	20.1 ± 8.5	0.048 * (0.36)
	MF	15.9 ± 6.7	22.6 ± 9.6	0.082 (0.14)
	OE	27.2 ± 9.5	29.1 ± 10.1	0.697 (0.01)
**APA_RER_M** **(%/s)**	ESL	92.1 ± 33.3	43.0 ± 19.6	<0.001 * (0.46)
	ESR	78.0 ± 48.1	43.9 ± 22.0	0.041 * (0.15)
	MF	61.0 ± 31.0	48.0 ± 24.2	0.272 (0.05)
	OE	19.4 ± 9.2	9.8 ± 7.5	0.017 * (0.26)
**APA_RER_SD** **(%/s)**	ESL	38.0 ± 16.4	19.2 ± 6.8	0.004 * (0.51)
	ESR	29.5 ± 17.7	20.7 ± 8.9	0.171 (0.09)
	MF	27.8 ± 13.4	18.7 ± 9.1	0.084 (0.14)
	OE	8.6 ± 4.2	4.0 ± 3.6	0.015 * (0.26)

FLEX—flexion, L_FLEX—right lateral flexion, EXT—extension, E/F RAT—extension/flexion ratio, PRR—postural reflex responses, APA—anticipatory postural adaptations, LAT—latency, AMP—amplitude, ms—milliseconds, Nm—newton-meter, M—mean, SD—standard deviation, ES—effect size. Asterisks represents significant difference of observed muscles (ESL—left erector spinae, ESR—right erector spinae, MF—multifidus, OE—obliquus externus) between the groups (*p* < 0.05). RER was defined as a percentage of peak amplitude of a single task.
